# The Tip of the Iceberg: Clinical Implications of Genomic Sequencing Projects in Head and Neck Cancer

**DOI:** 10.3390/cancers7040879

**Published:** 2015-10-21

**Authors:** Andrew C. Birkeland, Megan L. Ludwig, Taha S. Meraj, J. Chad Brenner, Mark E. Prince

**Affiliations:** 1 Department of Otolaryngology—Head and Neck Surgery, University of Michigan Health Systems, Ann Arbor, MI 48109, USA; abirkela@med.umich.edu (A.C.B.); ludwigml@umich.edu (M.L.L.); stmahmoo@med.umich.edu (T.S.M.); chadbren@med.umich.edu (J.C.B.); 2 Comprehensive Cancer Center, University of Michigan Health Systems, Ann Arbor, MI 48109, USA

**Keywords:** HNSCC, targeted therapy, TCGA, genomic, cancer, precision medicine, personalized medicine

## Abstract

Recent genomic sequencing studies have provided valuable insight into genetic aberrations in head and neck squamous cell carcinoma. Despite these great advances, certain hurdles exist in translating genomic findings to clinical care. Further correlation of genetic findings to clinical outcomes, additional analyses of subgroups of head and neck cancers and follow-up investigation into genetic heterogeneity are needed. While the development of targeted therapy trials is of key importance, numerous challenges exist in establishing and optimizing such programs. This review discusses potential upcoming steps for further genetic evaluation of head and neck cancers and implementation of genetic findings into precision medicine trials.

## 1. Background

Head and neck squamous cell carcinoma (HNSCC) remains a disease with very poor outcomes, particularly in advanced and recurrent tumors [1]. In the past few years, monumental advances, led by The Cancer Genome Atlas (TCGA), have been made in sequencing HNSCCs [2,3,4]. These studies have provided valuable insight into the genetic processes regulating HNSCCs. However, the vast amount of information generated by these studies has outpaced our ability to interpret and apply the data. Moving forward, we will need to continue to collect and sift through these data to identify drivers of tumorigenesis, biomarkers for prognosis and targets for new therapy. Additionally, institutional and national guidelines should be established on criteria for when and how to employ our expanding repertoire of targeted therapy agents.

## 2. Further Need for Genetic Analysis and Follow-Up Genomic Studies

Initial whole exome sequencing studies by Stransky *et al.* [3] and Agrawal *et al.* [4] identified commonly-mutated genes in HNSCC. This was expanded upon with the TCGA, with an initial cohort of 279 HNSCC patients [2]. Whole exome sequencing of additional cohorts has added to these data [5,6]. Information from these populations has highlighted common genetic aberrations in HNSCC, many of which had been previously established (e.g., *TP53*, *PIK3CA*), and some of which were newly identified (e.g., *NOTCH1*; Table 1). Useful databases cataloguing clinical, mutation, copy number and expression data from these studies are publically available and were used for analysis in this manuscript [7,8].

**Table 1:** Mutation rates in HNSCC from whole exome sequencing studies. The most commonly-mutated genes of interest across these studies are listed.

Gene	Stransky *et al.* [3] *n* = 74	Agrawal *et al.* [4] *n* = 32	TCGA [2] *n* = 279	Pickering *et al.* [5] *n* = 44	Total *n* = 429
*TP53*	46 (62%)	22 (69%)	204 (73%)	31 (70%)	303 (71%)
*FAT1*	9 (12%)	0 (0%)	64 (23%)	8 (18%)	81 (19%)
*CDKN2A*	9 (12%)	0 (0%)	63 (23%)	2 (5%)	74 (17%)
*PIK3CA*	6 (8%)	3 (9%)	58 (21%)	3 (7%)	70 (16%)
*NOTCH1*	9 (12%)	4 (13%)	52 (19%)	9 (20%)	74 (17%)
*CASP8*	6 (8%)	1 (3%)	24 (9%)	4 (9%)	35 (8%)

### 2.1. Limited Data on Early Stage, HPV+, Non-Caucasian Patients

While these initial sequencing studies have been immensely useful, there are some limitations with the selected cohorts. The initial TCGA cohort (*n* = 279) is primarily Caucasian (87%), primarily male (73%), with a smoking history (79%), older (mean age 61) and primarily negative for human papillomavirus (HPV−; 87%). Similarly, the Stransky and Agrawal cohorts generally characterized older male HPV−, smoker cohorts. Thus, there may be limitations on extrapolating data to other subsets of patients (women, younger patients and other ethnicities), as different mutational drivers may be more prevalent in other cohorts. In addition, the majority of tumors in the TCGA, Stransky and Agrawal cohorts were advanced in stage, with a limited number of early-stage (I/II) tumors for analysis (21%, 8% and 9%, respectively). There has been some follow-up effort into looking at younger and non-smoking populations [5], but the overall number of patients remains underpowered for definitive analysis.

In addition, these initial cohorts contained few HPV+ patients (13%–15%) [2,3,4,9], making interpretation of mutational patterns in these patients limited. Subsequent studies have investigated additional HPV+ specimens with targeted genetic sequencing [10,11], with some initial analysis suggesting increased mutation rates in *PIK3CA* and dysregulations in *FGFR3* [2,9,10,11]. Nevertheless full analysis of HPV+ tumors remains difficult to interpret due to limited study power.

Thus, further investigation into the mutation patterns of young patients, non-smokers, non-Caucasian patients and HPV+ tumors may provide insight into key regulatory and targetable differences between these cohorts and the traditional and well-studied older, Caucasian, tobacco- and alcohol-using, HPV− HNSCC cohort. Additionally, further investigation of HPV integration patterns into the genome may illustrate additional genes or gene products that may be aberrantly regulated or lead to altered genomic amplification and rearrangement in HPV+ HNSCC [9,12].

### 2.2. Subsequent Sequencing Studies

A question going forward in capturing additional tumors and patients for sequencing is to what degree we should sequence tumor specimens. Are panels of frequently-mutated and actionable genes sufficient, or is whole exome/genome sequencing a more useful tool in HNSCC? Notably, there is a high mutational variability and rate (both with copy number variations and sequence mutations) in HNSCC [2]. Nevertheless, core dysregulated pathways and actionable targets are already being identified (Table 1), justifying more focused and cost-effective sequencing panels that may yield similar information with less noise. Some investigators have performed targeted panels for sequencing analysis on subsets (200–600+) of actionable and potentially prognostic genes in HNSCC [10,11], with similar overall findings as whole exome studies.

### 2.3. Genetic Biomarkers

**Figure 1:**
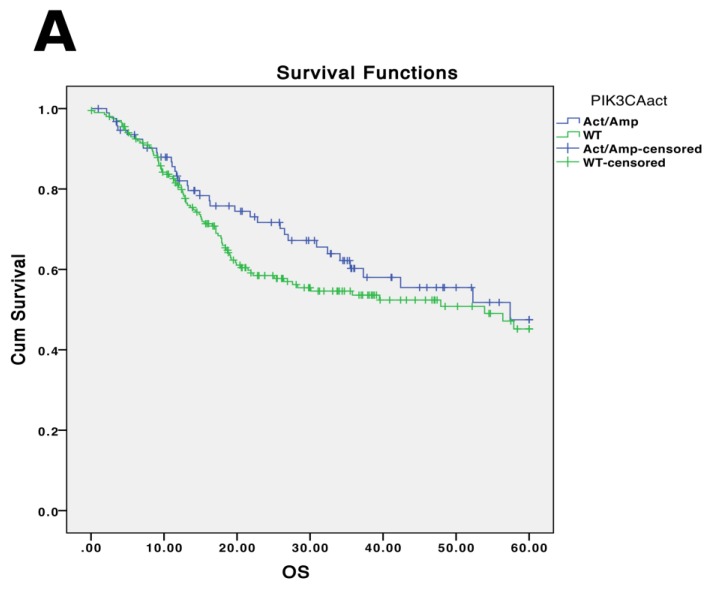

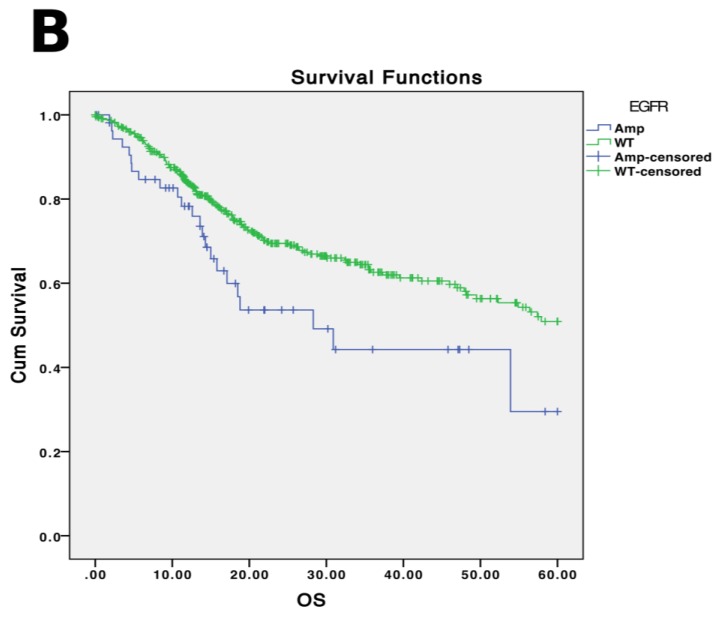
Five year overall survival (x-axis in months) and genetic status from TCGA. *PIK3CA*-activating mutations and amplifications do not correlate with worse overall survival (*p* = 0.292) (**A**) in the TCGA cohort, while *EGFR* amplifications do correlate with worse overall survival (*p* = 0.016) (**B**). Survival trends are consistent when analyzing all stages and when analyzing advanced-staged (III/IV) tumors specifically (data not shown).

Historically, protein expression biomarkers have been used to develop prediction algorithms for disease outcomes and response to therapy [13,14]. With the large volume of data available from the TCGA and accompanying studies, we can begin to identify genetic predictors of disease outcomes. Previous studies have associated *PIK3CA* mutations, *TP53* mutations and *EGFR* copy number amplifications with worse survival in HNSCC [6,10,15,16]. However, there is a lack of further significant analysis into additional genetic mutational predictors of survival or clinical phenotypes. Moreover, the data conflict over whether certain genetic mutations correlate with survival. While some studies have demonstrated significantly worse prognosis with *PIK3CA*-activating mutations, this is not evidenced when analyzing TCGA data (*p* = 0.292; Figure 1A; Table 2). *EGFR* amplifications, however, do correlate with overall survival in the TCGA cohort, consistent with previous studies (*p* = 0.016; Figure 1B, Table 2). Expanding analyses to multiple genes within a pathway, or multiple genes affecting similar cellular functions, may provide more insight into key aberrations and genetic signatures that may be associated with clinical outcomes. Further thorough collection of clinical data in conjunction with sequencing and continual follow-up of clinical outcomes of sequencing cohorts will provide valuable information into genetic biomarkers.

**Table 2:** Genetic alterations and overall survival in the initial TCGA cohort in months (mo). In the initial TCGA cohort (*n* = 279), individual genetic alterations associated with worse overall survival include *TP53* and *EGFR*, consistent with previous studies. Notably, this analysis does not stratify the type of mutation (activating/amplification *vs.* loss-of-function/deletion *vs.* benign).

	Altered Median OS (mo)	WT Median OS (mo)	Log-Rank *p*-Value
*TP53*	19.2	65.8	0.04
*FAT1*	18.8	26.4	0.80
*CDKN2A*	17.2	52.3	0.24
*PIK3CA*	28.3	21.9	0.41
*NOTCH1*	18.0	28.3	0.49
*CASP8*	18.8	26.4	0.31
*EGFR*	17.2	30.0	0.03

### 2.4. Insight across Multiple TCGA Cohorts

Additional TCGA cohorts can be used to compare mutational profiles between HNSCC and other tumor types [17,18,19,20]. Comparisons may be most relevant between HNSCC and other squamous cell carcinomas, including lung and cervical squamous cell carcinomas (Table 3 and Table 4). Interestingly, HNSCC overall has a higher mutation rate and, in particular, higher prevalence of mutations in *FAT1*, *NOTCH1* and *CASP8* (*p* < 0.05; Table 3). In addition, HNSCC has higher rates of 11q13 amplification (which contains *FGF3*/*4*/*19* and *CCND1*) in comparison with other epithelial squamous cancers (*p* < 0.001; Table 4).

**Table 3:** Mutations in across TCGA studies. Comparison of mutational frequencies across the multiple genes in HNSCC, cervical, nasopharyngeal, esophageal and lung squamous cell carcinoma. Mutation frequency in *FAT1*, *NOTCH1* and *CASP8* in HNSCC is significantly higher (*p* < 0.05) in comparison to other cohorts of squamous or similar cancers.

	HNSCC (*n* = 279)	Cervical (*n* = 171)	Lung (*n* = 178)	Esophageal (*n* = 88)	Nasopharyngeal (*n* = 56)
*TP53*	73.1%	2.9%	93.3%	83.0%	12.5%
*FAT1*	22.9%	4.1%	14.6%	4.5%	3.6%
*CDKN2A*	22.6%	1.2%	18.0%	4.5%	0.0%
*PIK3CA*	20.8%	26.3%	15.7%	4.5%	1.8%
*NOTCH1*	18.6%	6.4%	8.4%	9.1%	3.6%
*CASP8*	8.6%	4.7%	1.7%	0.0%	1.8%

**Table 4:** Copy number variations (CNV) across TCGA studies. Comparison of CNV frequencies across genetic loci in HNSCC, cervical and lung squamous cell carcinoma. Amplification of the 11q13 locus is significantly higher in HNSCC in comparison with cervical and lung SCCs (*p* < 0.001).

	HNSCC (*n* = 522)	Cervical (*n* = 295)	Lung (*n* = 501)
9p21 (*CDKN2A*, *CDKN2B*)	27.7%–31.3%	0.3%	23.0%–27.6%
	Deletion	Deletion	Deletion
11q13 (*FGF3*/*4*/*19*,*CCND1*)	23.4%–24.2%	2.9%–10.4%	14.1%–14.3%
	Amplification	Amplification	Amplification
3q28 (*TP63*, *EIF4A2*, *FGF12*)	18.7%–21.5%	19.2%–21.4%	38.9%–41.9%
	Amplification	Amplification	Amplification
3q26 (*SOX2*, *PIK3CA*)	18.7%%–20.8%	19.5%–21.1%	43.5%–48.0%
	Amplification	Amplification	Amplification
8q24 (*MYC*, *PLEC*, *EPPK1*)	8.3%–12.3%	2.6%–4.9%	6.9%–10.5%
	Amplification	Amplification	Amplification

Squamous cell cancers independently have been identified to have similar molecular aberrations across cancer types, including *TP53*, *PIK3CA*, *CDKN2A*, *SOX2*, *CCND1* and *NOTCH1* [21], which is consistent with the mutational profile of HNSCC. Further stratifying oncogenic signatures [22] for HNSCCs and identifying further subgroups of mutational patterns in HNSCC will be valuable in providing greater insight into varying mechanisms of pathogenesis and, potentially, treatment response.

## 3. Further Need for Tumor Stratification

Despite our advances in tumor sequencing, various issues have not been fully investigated in regards to addressing tumor heterogeneity and identifying driver mutations.

### 3.1. Driver vs. Passenger Mutations

Despite our advances in next-generation sequencing and the large amount of information given to us, we have not been able to fully assess which of these mutational events are the “drivers” and which are the “passengers” [23]. While many predictive tools exist to determine the functional effects of specific mutations [24], they remain imperfect. Furthermore, it can be difficult to assess the biologic effect of copy number variations as drivers of disease. Tumor sequencing heterogeneity has provided some insight into early driver mutations that are common to all tumor cells within a population, *versus* later passenger mutations that may appear in only subsets of tumor cells [25,26,27]. Thus, performing sequencing on multiple biopsy sites of a tumor may have added value in phylogenetically-identifying common core driver mutations that are derived early in a tumor from subsequent additional passenger mutations [26]. However, such studies would not distinguish late additional driver mutations. Functional follow-up studies remain of paramount importance to validate suspected mutational drivers and to identify targetable genetic options for therapy.

Further analysis of epidemiologically low risk patients in which generally acknowledged causes of HNSCC are absent (young, non-smoking, non-drinking, HPV−) [5,28] may provide insight into genetic drivers of HNSCC, as mutation rates will likely be lower in this cohort. Thus, any genetic mutations or copy number variants have a higher probability of being driver mutations. Furthermore, identifying and sequencing patients with distinct second primary tumors can highlight dysregulated pathways common to both tumors to increase the likelihood of identifying a “driver” mutation.

### 3.2. Early vs. Late-Stage Mutations

Identification of early mutational events and sequences leading to initial dysplasia and carcinoma *in situ* will be invaluable to determine if the genetic signatures between dysplasia and frank carcinoma and early and late cancers is different. By uncovering sequential mutational status, mutational pathways and steps to carcinogenesis in HNSCC can be fully elucidated. Although Hanahan and Weinberg famously described the hallmarks of carcinogenesis [29], specific mutational progressions driving cancer formation from normal and dysplastic tissue have yet to be identified in HNSCC. Further collection of surrounding normal tissue, dysplastic tissue and different tumor subpopulations within a heterogeneous tumor can give insight into the steps of genetic progression [26]. In addition, the collection and investigation of surrounding dysplastic tissue/carcinoma *in situ* and early-stage tumors would be highly beneficial, as the overall mutational burden would likely be less in these early-stage tumors. As a result, there may be an increased likelihood of the identification of driver mutations as the noise from passenger mutations is decreased. As tumors advance from early to late stages, sequencing primary tumor biopsies at different time points can provide informative results as to the drivers of tumor progression. Additionally, sequencing nodal and distant metastases for comparison to the primary tumor may highlight drivers of cancer invasiveness and metastasis.

Investigation into the role of the tumor microenvironment may provide insight into cell-extrinsic drivers from dysplasia/carcinoma *in situ* to early stage to advanced stage tumors. This includes immune surveillance, local growth/stimulatory factors, extracellular matrices and angiogenic factors. Expression profiling of the tumor microenvironment can elucidate the extrinsic modulators of tumor progression, and the interaction this extrinsic milieu has with specific tumor mutations. As discussed below, early investigation into immune modulators has shown impressive results.

### 3.3. Tumor Temporal and Spatial Heterogeneity

It is well established that tumors contain heterogeneous populations of cells [30]. Importantly, different subsets of tumor cells may derive *de novo* mutations, leading to a tumor with heterogeneity in genetic mutations [26]. Different tumor cell clonal lines may subsequently develop with significantly different behavior and different druggable targets. Thus, the evaluation of mutations in a single biopsy of a tumor may not be indicative of the whole tumor, but rather a subset of tumor cells. Mroz *et al.* recently established increased mutant allele tumor heterogeneity as a predictor of poor clinical outcomes [31,32]. Further expanding on this concept to identify specific mutational profiles that correlate to poorer outcomes as genetic biomarkers may provide further benefit in employing genomic data for clinical paradigms. Tumor spatial heterogeneity models are increasingly being studied in order to account for the development of tumor subpopulations with distinct genetic mutations [25]. Moving forward, tumor microdissection and sequencing may provide useful information in regards to common underlying driver mutations within a single tumor, as well as aggressive subclonal driver mutations.

As discussed above, temporal tumor evolution and increased heterogeneity remains a challenging hurdle for clinical consideration of sequencing results. The TCGA and similar sequencing studies to date have been generated from single biopsies at a single time point. As subclonal populations inevitably arise in tumors, further temporal monitoring of tumors’ genetics will be valuable to identify additional altered targetable genes within tumor subpopulations.

As single-cell sequencing becomes increasingly feasible [33] and temporal and spatial analytics of driver mutations improve [25], employing these technologies to account for tumor heterogeneity may lead to increased success in matching ideal targeted agents for patients at the right time. Until then, trials of targeted therapy against tumors that grossly carry targetable mutations will remain in place.

### 3.4. Cancer Stem Cells

A key component of tumor heterogeneity may derive from the presence of cancer stem cells (CSC) or tumor-initiating cells. Multiple studies have demonstrated that this subset of tumor cells has increased invasiveness, metastatic potential and resistance to chemotherapy and radiation [34,35]. However, genetic and expression signatures of CSCs, as well as drivers of increased CSC populations, have not been elucidated on a genome-wide scale at this time. The genomic mutational profile of this key subpopulation of cells may provide insight into their aggressive behavior and potential for targeting. Isolating these CSCs for specific mutational sequencing, copy number analysis and RNA expression profiles in comparison to the non-CSC tumor cells may elucidate the mechanisms governing the differences in behavior in this cell population. Additionally, CSC proportions within tumors vary greatly (from 0.4%–81%), with greater percentages of CSCs correlating with more aggressive tumors [36]. Correlating drivers of increased CSC percentages within tumors may uncover novel targets for inhibiting CSC-specific growth and proliferation.

### 3.5. Molecular Heterogeneity

Further complicating analyses of genomic studies are conflicting data on the role of aberrantly-regulated genes identified in TCGA and other studies. A key finding of initial exome sequencing studies was the identification of inactivating mutations in the *NOTCH* pathway in HNSCC, suggesting a tumor suppressor status [2,3,4]. However, subsequent studies and analyses have correlated *NOTCH1*-activating mutations with HNSCC, suggesting a bimodal function of *NOTCH1* [37]. Thus, developing *NOTCH* as a potential therapeutic target is difficult, as both over- and under-activity of the pathway are associated with carcinogenesis in the head and neck.

**Figure 2:**
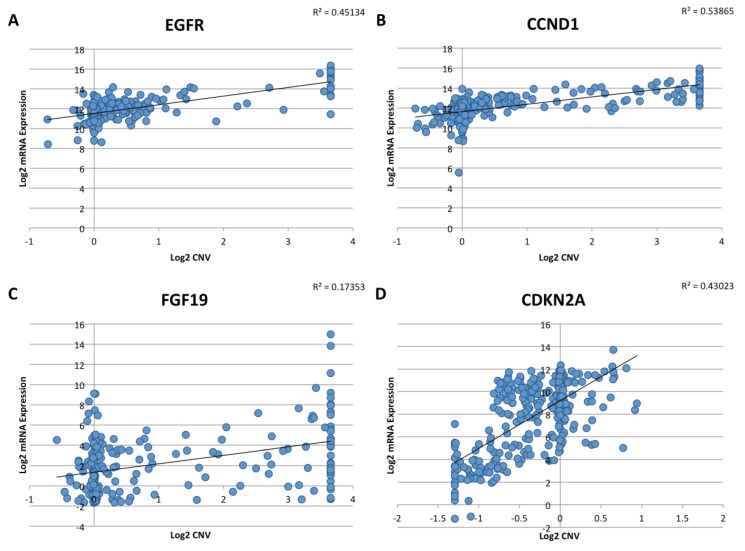
Copy number variation and mRNA level in TCGA. The mRNA level does not necessarily correlate with DNA copy number. As an example, mRNA levels of several commonly amplified (*EGFR*, *CCND1*, *FGF19*) (**A**–**C**) and deleted (*CDKN2A*) (**D**) genes show poor correlation with copy number in the TCGA dataset. Data generated from cBioPortal [7,8]. Of note, the copy number caller on cBioPortal has upper and lower limits.

Additionally, molecular heterogeneity exists between genomic amplification and expression of genes. In TCGA, gene amplification does not always correlate with RNA expression levels (Figure 2). While some genes demonstrate significant copy number amplifications or deletions, actual mRNA expression levels do not fully correspond with gene copy number. Potentially, epigenetic and non-coding regulators of transcription may play a role in final gene product expression levels. Thus, more specific accounting of the expression status of a gene (either through RNA or protein levels) may be more valuable in characterizing pathway dysregulations in HNSCC.

### 3.6. Tissue-Specific Mutational Profiles

**Figure 3:**
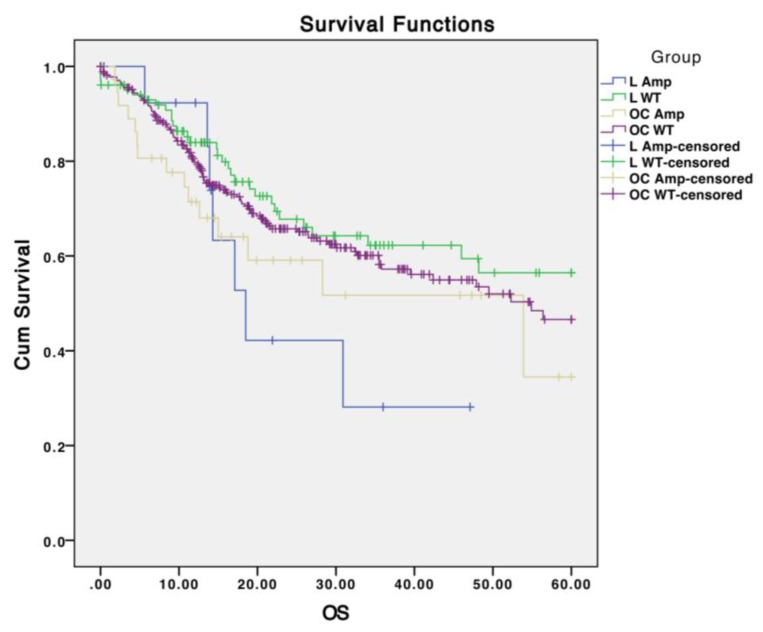
Survival based on tumor subsite, *EGFR* amplification status. Five-year overall survival (x-axis in months) from 522 TCGA patients with CNV data on *EGFR* amplification in oral cavity (OC) and laryngeal (L) SCCs. A trend to worse overall survival based on *EGFR* amplification is seen with laryngeal SCCs (log-rank *p* = 0.08), but not oral cavity SCCs (log-rank *p* = 0.263).

Stratification of HNSCCs into subsites and analysis of mutational profile differences across different subsites has been limited. While studies suggest different mutation rates between oropharyngeal tumors and other tumors (Table 5), this is thought to be in part driven by HPV status. Further stratification of tumor sites could elucidate different aberrant pathways regulating carcinogenesis and survival. For instance, although *EGFR* amplifications do not strongly correlate with survival in oral cavity SCCs, they trend to worse survival in laryngeal SCCs (Figure 3). Further collection of samples to determine whether the presence of *EGFR* amplification is an important biomarker specifically in larynx SCCs and investigation into biologic differences between larynx and oral cavity SCCs should be performed. Moreover, amplifications in 3q26 (which include *PIK3CA*) are found significantly more frequently in laryngeal SCCs in comparison to oral cavity SCCs (*p* < 0.01; Table 6), and mutations in *PIK3CA* trend towards more frequent in laryngeal SCC in comparison to oral cavity SCC (*p* = 0.17; Table 5). Further analysis of site-specific mutational profiles in conjunction with increasing tumor numbers of underrepresented sites (oropharynx, larynx, hypopharynx) should be performed to capture different mutational profiles and drivers across HNSCC subsites.

**Table 5:** Mutation rates by subsite from TCGA. Subsites defined from the initial 279 patients in TCGA. Key frequently-mutated genes are highlighted. OC = oral cavity; OP = oropharynx; L = larynx.

	OC (*n* = 172)	OP (*n* = 33)	L (*n* = 72)
HPV+	7.0%	66.7%	1.4%
*TP53*	75.6%	27.3%	88.9%
*FAT1*	26.2%	12.1%	19.4%
*CDKN2A*	25.6%	6.1%	23.6%
*PIK3CA*	17.4%	30.3%	25.0%
*NOTCH1*	21.5%	6.1%	18.1%

**Table 6:** Copy number variants by subsite from TCGA. Subsites defined from the initial 279 patients in TCGA. Frequently-altered amplicons, with key genes in each amplicon, are noted. Significantly higher rates of amplification of the 3q26 locus are seen in larynx specimens in comparison to oral cavity specimens (*p* < 0.01). OC = oral cavity; OP = oropharynx; L = larynx.

	OC (*n* = 172)	OP (*n* = 33)	L (*n* = 72)
9p21 (*CDKN2A*, *CDKN2B*)	26.2%–28.5%	18.2%	30.6%–31.9%
	Deletion	Deletion	Deletion
11q13 (*FGF3*/*4*/*19*, *CCND1*)	25.0%	24.2%	36.1%
	Amplification	Amplification	Amplification
3q28 (*TP63*, *EIF4A2*, *FGF12*)	12.2%–12.8%	27.3%–30.3%	30.6%–34.7%
	Amplification	Amplification	Amplification
3q26 (*SOX2*, *PIK3CA*)	12.8%–13.4%	27.3%–30.3%	34.7%–37.5%
	Amplification	Amplification	Amplification
8q24 (*MYC*, *PLEC*, *EPPK1*)	9.3%–11.0%	9.1%–12.1%	12.5%–16.7%
	Amplification	Amplification	Amplification

## 4. Challenges to Targeted Therapy

The next frontier in using the data generated from these sequencing studies is to identify and study targetable mutations. Encouragingly, there is an ever-increasing number of drugs that target many of the dysregulated genes and genetic pathways in HNSCCs. Many of these are either FDA-approved or in clinical trials for other tumors, and a large number of these drugs is in clinical trials for HNSCC, as well [38]. Despite these advances, though, many obstacles remain in this arena.

### 4.1. What is Actionable?

One goal of NGS studies is to identify “actionable” results. As discussed above, mutational “drivers” that are targetable are ideal candidate genes for targeted therapy. However, in many cases, multiple potential “actionable” mutations exist, and it is difficult to elucidate the critical driver mutations. Thus, questions arise over potential hierarchies of mutational events to target. Should all “actionable” results be targeted? Can a tumor have multiple drivers that should be targeted? In what sequence should these drivers be targeted? Studies should be designed to determine which genetic aberration, or combination of genetic aberrations, should be targeted when encountered.

Further confounding this analysis is our experience with cetuximab and *EGFR* status. Although cetuximab is approved for treatment in HNSCC [39,40], it is used independent of any *EGFR* evaluation, including mutation and amplification status. Interestingly, research to stratify responders to cetuximab based on *EGFR* status has not yielded any predictive results [39,40]. Thus, the question arises: should we evaluate subsequent targeted agents based on individual tumor aberration, or should targeted agents be universally applied, irrespective of mutational status?

Additionally, as tumors progress over time and develop subclonal populations, nodal and distant metastases, actionable targets may evolve. Tumors may acquire resistance mechanisms to targeted agents [41,42], and new mutational drivers may arise. Thus, it may be necessary to continually reassess the genetic landscape of tumors undergoing targeted therapy.

### 4.2. Creating Targeted Therapeutics

A major challenge in the development of targeted antibodies and small molecule inhibitors is that the majority of the current targets work to inhibit oncogenes. A large number of mutations identified in sequencing studies, however, are loss of function mutations in tumor suppressor genes (e.g., *TP53*). A greater challenge will be to restore functional balance to affected pathways in these cases. Potentially, this may be achieved through either restoration of gene function or inhibition of unchecked pathways [43,44].

An avenue of exploration that can lead to earlier implementation of targeted therapy in HNSCC is to identify genetic targets that have therapeutics already developed for use in other cancers. As the biosafety profiles of such targeted drugs have been established in these cases, use in HNSCC may be more easily attained. Indeed, personalized medicine paradigms are exploring the treatment of cancer based on mutational profiles rather than organ site [45].

### 4.3. Development of Resistance to Monotherapy

Targeting HNSCC may require a combination of therapies, as treatment with monotherapy may select for resistant clonal populations, which will then repopulate the tumor with resistant cells. Thus, continual reassessment of the molecular signature of tumors may need to be performed, as this may constantly be in flux. For instance, melanomas with the BRAF V600E mutation are responsive to vemurafenib [46]. However, these tumors almost invariably develop resistance to vemurafenib, requiring further investigation into resistance mechanisms and alternative targets [47]. Combination therapies of targeted inhibitors, or the addition of traditional chemotherapy and radiation, may circumvent this resistance [38].

### 4.4. Organ-Specific Response to Therapy

Similar genetic aberrations may not respond similarly across different organs. A classic example of this is with *HER2*-positive ovarian cancers. While *HER2* amplification is treatable with Herceptin (trastuzumab) in breast and gastrointestinal cancers, *HER2* positive ovarian cancers do not respond as well to trastuzumab [48]. Thus, targeted agents that may have benefits in other organ systems may not show survival benefit in HNSCC. Moreover, agents that may be beneficial in one head and neck subsite may not be applicable in other subsites (e.g., agents may have improved outcomes in oropharynx cancers compared to larynx cancers).

### 4.5. Targeting Drivers of Cancer Stem Cells

Response to targeted agents between CSCs and remaining tumor cells remains a little understood research avenue. Interestingly, aberrations in canonical CSC markers (CD44, CD133 and ALDH, among others) were not identified in genetic sequencing studies [2,3,4]. Given the importance of CSC prevalence within a tumor in association with tumor aggressiveness [36], identifying genetic drivers of CSC frequency within tumor populations may prove to be a fruitful avenue of exploration.

## 5. Translation to Clinical Targeted Therapy Paradigms

### 5.1. When to Use Targeted Therapy

Institutional and national guidelines will need to be established based on when to employ targeted therapeutic agents. Currently, treatment algorithms exist for cetuximab (Erbitux), but no other targeted agents in HNSCC. A question for timing includes the potential use of targeted agents as induction therapy to evaluate for treatment response, similar to induction chemotherapy paradigms [49]. In non-responding tumors, traditional therapy and management could then be applied.

Which patient populations are ideal for drug investigation also remains undefined. It is logical to enroll patients with recurrent or advanced tumors into targeted therapy trials, particularly if other treatment avenues have been exhausted (surgery, chemotherapy and radiation). In cases where palliative therapy may be the only other option, targeted therapy may provide a chance for prolonged survival. However, given the poor prognosis of these patients and the aggressive biology of these tumors, otherwise potentially useful targeted agents may not demonstrate great efficacy. A more meaningful clinical response could potentially be achieved in cases of less aggressive and earlier tumors, potentially in organ-preserving and induction therapy trials. Overall, targeted agents may provide benefits in a variety of clinical scenarios, including induction/neoadjuvant, concurrent, adjuvant, recurrent and palliative settings. To date, many of these potentially beneficial clinical paradigms remain unexplored.

### 5.2. Incorporating Targeted Therapy with Other Treatment Paradigms (Immunotherapy, Surgery, Chemoradiation)

In conjunction with the question of when to use targeted therapy, another question is with what other treatment paradigms should targeted therapy be employed. Clinical trials have demonstrated a benefit for cetuximab in addition to cisplatin or radiation in specific instances [39,40]. Similar clinical trials will inevitably need to be performed as we identify subsequent targetable therapeutics. As an example, the University of Michigan is incorporating an inhibitor of Bcl-2 (AT101) in combination with induction chemotherapy (NCT01633541).

Recent immune checkpoint blockade studies regarding a PD-1 inhibitor (pembrolizumab) have shown impressive survival benefit, particularly in comparison to cetuximab [50], indicating that newer targeted therapeutic agents can provide survival benefits. Furthermore, new clinical trials are studying ipilimumab (another immune checkpoint blockade agent FDA-approved for melanoma) in conjunction with cetuximab and radiation therapy (NCT01935921). Such combinations of immunotherapy with surgery, chemotherapy and radiation may demonstrate further efficacy of these agents.

Questions as to the various combinations between chemotherapy, radiation and targeted therapy remain. The possibility of de-escalation of therapy, limiting the number of treatment modalities, may exist if patients respond particularly well to a targeted therapy. Moreover, combinations of targeted therapy may prove to be more successful than single targeted therapy agents in order to more fully address codependent pathways [38].

### 5.3. Employing Agents Approved in Other Cancers

An early applicable option for HNSCCs is to test agents with proven benefit in other cancers. Many of the dysregulated genes identified in sequencing studies already have targeted agents and similar mutations to other cancers. For instance, *HER2* amplifications are important in breast and gastrointestinal cancers, as they are targetable with trastuzumab (Herceptin), with a resultant significant survival benefit. Interestingly, *HER2* amplifications are present in a subset of HNSCCs [2], suggesting a potential role for HER2 targeted therapy in HNSCCs [51].

### 5.4. Precision Medicine Clinical Trials

Several groups have already enrolled patients with metastatic or recurrent cancers into clinical sequencing trials, including Gustave Roussy (France), Weill Cornell and the University of Michigan (Mi-ONCOseq) [52,53,54]. Each trial was able to successfully discover actionable mutations in a subset of patient molecular profiles (up to 40% in HNSCC). Gustave Roussy had the highest rate of success, with 10/68 (15%) of patients having sequencing that informs therapeutic decisions [52]. These actionable cases are lower than expected, and a common problem for the majority of non-actionable patients was the advanced stage of the cancer. While groups reported an average turnaround of sequencing results of four weeks, a subset of patients were already deceased or had advanced beyond eligibility [52,53]. Furthermore, there were several cases where sequencing results recommended specific targeted therapies, but clinical trials were not available or accessible at the time [54]. However, notably, dramatic successes were seen in these initial cohorts (e.g., Cornell reported a patient with metastatic urothelial carcinoma with a *HER2* amplification, a rare aberration in this tumor, that had a complete response when given targeted HER2 therapy). Each trial was able to demonstrate success in informing patient therapeutics, even if on a smaller scale than originally expected. Currently, Mi-ONCOseq has expanded from a pilot study and has enrolled several hundred patients with results pending. These results indicate the potential for clinical sequencing trials and highlight the challenges that still need to be overcome.

Learning from these initial studies, specific adjustments may be made to clinical trial paradigms to enhance patient capture, and potentially clinical success. For instance, current standards for enrollment are for recurrent and metastatic disease. Screening for these patients alone may yield skewed poor outcome data, as aggressive and otherwise resistant diseases are being selected. The biologic benefit of targeted therapy may lie with earlier-stage or less aggressive tumors, where targeted agents may be less morbid than current standards of care (surgery, nonspecific chemotherapy and radiation). Another method of studying the effects of targeted therapy is in neo-adjuvant settings. Induction chemotherapy paradigms have established means of stratifying tumors for organ preservation, and targeted therapy can follow in a similar fashion. Creating induction targeted therapy trials may lead to successful organ-preservation courses of care. Finally, use of targeted therapy may be most readily applied in adjuvant settings.

The temporal and spatial aspects of tumor development must be increasingly taken into account as we aim to increase our specificity and success of personalized medicine and targeted trials. Going forward, trials will have to increasingly take into account tumor subclonal temporal evolution and tumor heterogeneity. Investigating the use of targeted therapeutics against targets that exist in all tumor biopsy sites, *versus* subclonal populations, may highlight the need for combinations of targeted agents to address different tumor subpopulations. Performing biopsies for sequencing at regular intervals may also assess if aggressive subclonal populations with new, targetable mutations have arisen from the parent tumor. This may be especially prudent in tumors that initially respond to targeted therapy, before relapse [41,42].

### 5.5. Precision Medicine Tumor Boards

The numerous challenging concepts and continually-evolving field of cancer genomics and personalized medicine dictate the need for institutional precision medicine tumor boards and national guidelines. The National Cancer Institiute (NCI), interestingly, has launched NCI-Molecular Analysis for Therapy Choice (MATCH), a program that aims to dictate tumor treatment with targeted therapy based on molecular and genetic characteristics, rather than anatomic tumor site [45]. Already, numerous tertiary care centers are developing precision medicine tumor boards for specific tumors, including HNSCCs. For instance, the Michigan Otolaryngology Sequencing (MiOTOSeq) program at the University of Michigan is aimed at incorporating genetic evaluation as part of the initial workup and decision-making process at a multidisciplinary head and neck tumor board. These tumor boards are also ideal grounds on which new and future clinical trials involving targeted therapy in HNSCC may be evaluated. Notably, precision medicine tumor boards will need to be closely involved with genetic counselors and have strong awareness of the numerous ethical issues involved in personalized medicine, including, but not limited to, incidental findings, as well as autonomy *vs.* beneficence [55].

## 6. Conclusions

We are in an exciting era for personalized medicine and targeted therapy. As much as we have advanced in uncovering the underlying genetics driving HNSCC, we have just uncovered the tip of the iceberg. Further analyses await us in interpreting current sequencing results, generating further sequencing data and harnessing this new information into meaningful therapy.
